# Case report: Desmoid fibromatosis diagnosed in a 27-year-old male after being mistaken for a gastrointestinal stromal tumour

**DOI:** 10.3389/fmed.2022.998473

**Published:** 2022-11-10

**Authors:** Larissa Albino, Yimeng Guo, Julinor Bacani, Cheryl Mather, Jan-Erick Nilsson, Levinus A. Dieleman

**Affiliations:** ^1^Department of Medicine, University of Alberta, Edmonton, AB, Canada; ^2^Division of Gastroenterology, University of Alberta, Edmonton, AB, Canada; ^3^Division of Anatomical Pathology, Department of Laboratory Medicine and Pathology, University of Alberta, Edmonton, AB, Canada

**Keywords:** gastrointestinal stromal tumour, GIST, desmoid tumour, desmoid fibromatosis, case report

## Abstract

Despite being distinct lesions, gastrointestinal stromal tumours (GISTs) and desmoid fibromatosis may appear similar on imaging when they involve the stomach wall or bowel. As a result, they may be confused with one another when initially diagnosed. This report aims to present a case where a desmoid tumour was mistaken for a gastric GIST in a 27-year-old gentleman despite extensive investigation prior to exploratory laparotomy, and why differentiation through pathology, with a focus on the immunohistochemistry profile, is key for proper prognostication and appropriate management, including timely investigation for associated diseases such as Familial Adenomatous Polyposis in patients with desmoid tumours.

## Introduction

The gastrointestinal tract can be affected by both epithelial and non-epithelial tumours. When considering non-epithelial tumours, gastrointestinal stromal tumours (GISTs) are the most common with an incidence of 7.5–15 cases per million per year (1, 2). They originate from interstitial cells of Cajal, also known as the “pacemaker cells” of the GI tract, and can arise anywhere within the GI tract, with the most common locations being the stomach (50–60%) and small intestine (20–30%). Less than 5% of GISTs are found within the omentum and mesentery. GISTs mainly affect adults, with the median age being 55–65 years (3, 4). Clinical presentation varies by tumour size, location, and aggressiveness, and can range from minimal symptoms to vague epigastric pain and early satiety. In severe cases, patients may present with acute bowel obstruction, volvulus, or infarction (3, 5–7). In addition, all GISTS have “uncertain malignant potential,” and it is very difficult to predict their likelihood of metastasis (1–3).

Another type of non-epithelial gastrointestinal tumour commonly mistaken for a GIST is the desmoid tumour, also known as desmoid fibromatosis. This type of tumour carries an incidence of 2–4 cases per million per year (8, 9). Desmoid tumours are most often found intra-abdominally in the mesentery but can occasionally be found peripherally in the extremities (8–10). They are often seen at sites of traumatic or surgical scarring but can also occur spontaneously (11, 12). Unlike GISTs, they tend to be diagnosed in a younger population with the median age being 34 years (13, 14). Although desmoid tumours are considered benign, they can become quite large and tend to be locally aggressive. Therefore, patients are often asymptomatic but can develop symptoms as the mass grows into or invades adjacent structures, such as joints or muscles (11, 12, 15–17).

There is a vast amount of literature describing the differences between GISTs and desmoid tumours. However, these distinct lesions appear similar on imaging, particularly when they involve the stomach wall or bowel, and therefore may be confused with one another at the time of initial diagnosis. Here, we present a case where a mesenteric desmoid tumour was mistaken for a GIST despite extensive investigation prior to exploratory laparotomy, and why differentiation is key for proper prognostication and appropriate management.

## Case report

**FIGURE 1:**
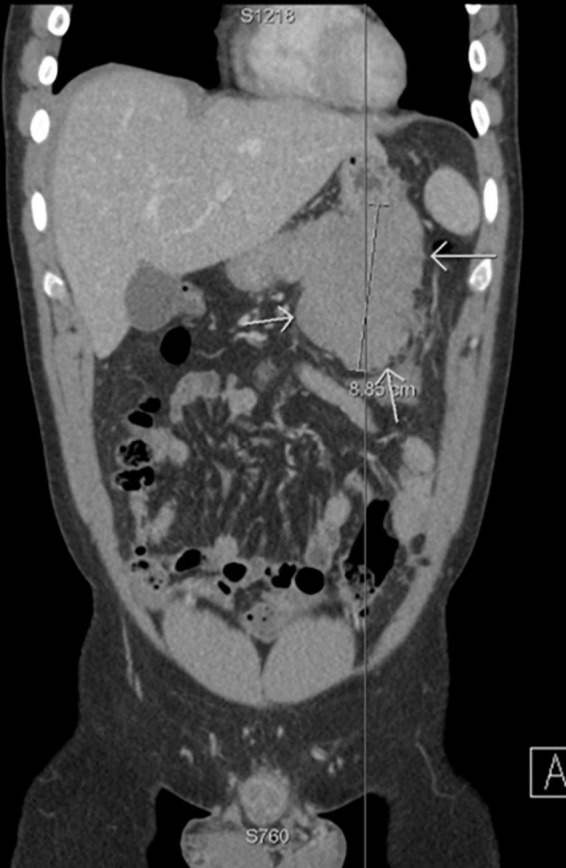
Coronal CT body demonstrating a large exophytic mass arising from the lesser sac of the stomach (indicated by white arrows), in keeping with a gastrointestinal stromal tumour (GIST).

**FIGURE 2:**
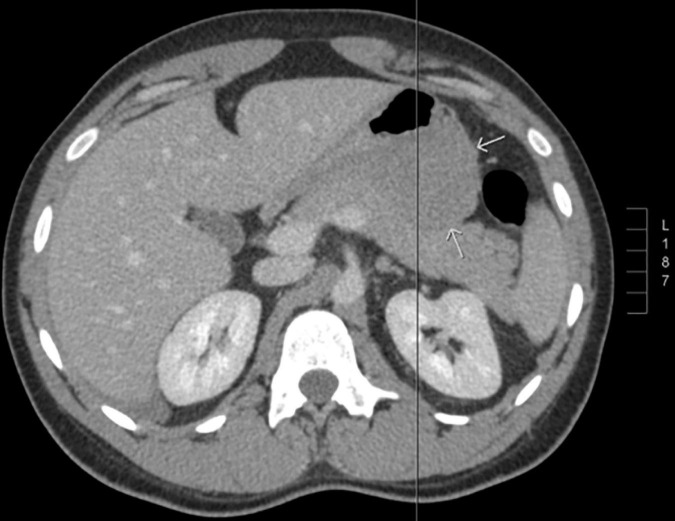
Axial CT body demonstrating a large exophytic mass arising from the lesser sac of the stomach (indicated by white arrows), in keeping with a gastrointestinal stromal tumour (GIST).

**FIGURE 3:**
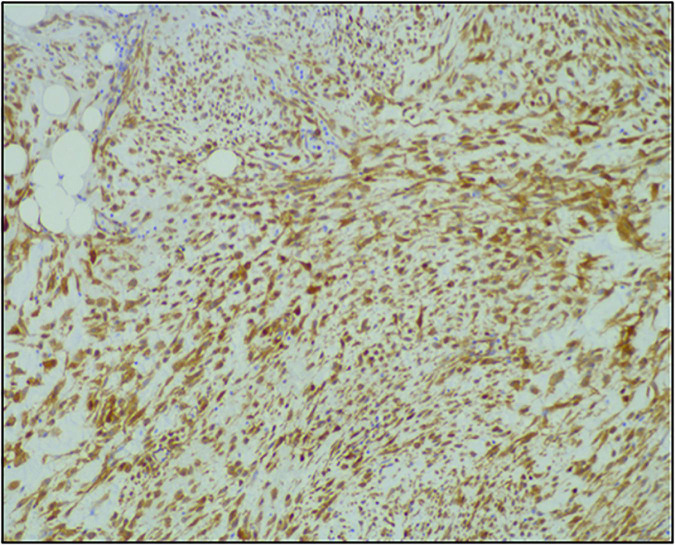
Immunohistochemistry staining of the resected mass demonstrating a low-grade spindle cell tumour, CD117/CD34/DOG-1 negative, with nuclear β-catenin staining, in keeping with desmoid fibromatosis.

**FIGURE 4:**
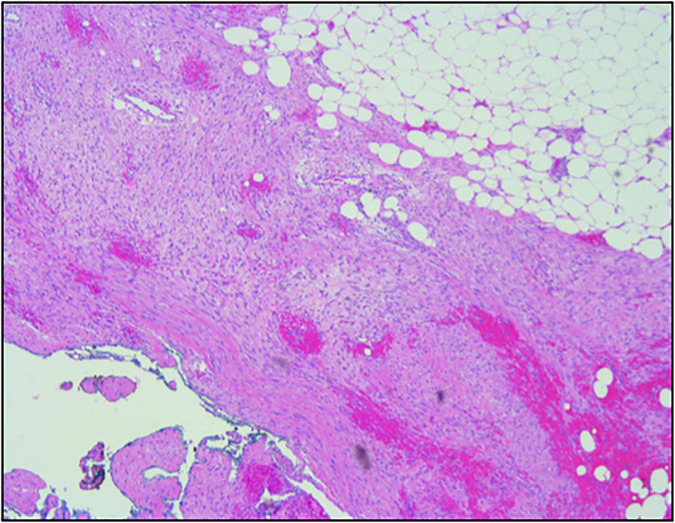
H&E staining at the margin of resection showing fibromatosis.

A previously healthy 27-year-old gentleman presented with a 2-day history of acute left-sided abdominal pain and postprandial fullness. He also noted a 2-month history of increased abdominal distension. Physical examination and laboratory investigations were unremarkable; however, he had a haemoglobin drop from 152 to 125 g/L over the span of a few hours. A CT-abdomen/pelvis demonstrated a large exophytic mass (5.2 cm × 6.0 cm × 9.5 cm) arising from the lesser sac of the stomach with a broad base of contact with the inferior aspect of the stomach and in close proximity to the transverse colon. There was also a moderate volume of hyperattenuating free fluid in the abdomen and pelvis. These findings were in keeping with a GIST with a possible component of intra-abdominal haemorrhage (Figures 1, 2). He was admitted and underwent further investigation with an endoscopic ultrasound, which demonstrated an exophytic mass arising from the muscularis propria (fourth layer) of the gastric wall and a central anechoic area consistent with haemorrhage. A biopsy was taken during the procedure but found to contain predominantly fresh blood. As these findings were most consistent with a GIST, an exploratory laparotomy was organised the following day. Despite previous imaging findings, a soft tissue tumour was found tethered to the mesentery of the transverse colon and the peritoneum of the antero-lateral abdominal wall, in immediate proximity to the greater curvature of the stomach. The mass had eroded into surrounding blood vessels, resulting in hemoperitoneum of roughly 700 cc. No obvious palpable mass was found in the stomach, small bowel, colon, or pancreas, and intraoperative esophagogastroduodenoscopy showed no mucosal lesion in the stomach. The hemoperitoneum was evacuated, the mass was resected, and a biopsy of the peritoneum was collected. Pathology identified a low-grade spindle cell tumour, CD117/CD34/DOG-1 negative with patchy cytoplasmic and nuclear β-catenin staining, in keeping with desmoid fibromatosis (Figures 3, 4). Ten days post-surgery, the patient underwent a colonoscopy to rule out Familial Adenomatous Polyposis (FAP), which is often associated with desmoid tumours. This study was limited by a seemingly fixed rectosigmoid colon, consistent with intraperitoneal desmoid fibromatosis. No findings suggestive of FAP were found up to the splenic flexure. He was discharged from hospital the following day with plans to repeat a CT-abdomen/pelvis in 1 year to assess for recurrence of the desmoid tumour.

CT-abdomen/pelvis was repeated which showed no evidence of local recurrence. However, an indeterminate 11 mm soft tissue density was seen in the left lower quadrant just deep to the abdominal wall with close proximity to the colon. It was unclear whether this was a new mass or if it was obscured on the prior imaging study. A follow up CT scan in 3–6 months was planned for re-evaluation. A repeat colonoscopy performed revealed three 5–9 mm sessile polyps that were removed *via* cold snare. Pathology demonstrated two tubular adenomas and one hyperplastic polyp. There was no evidence of FAP.

## Discussion

This case illustrates how GISTs and desmoid tumours are often mistaken for one another, despite a vast amount of literature describing the differences between the two. This is likely because the initial workup involves the use of poor diagnostic imaging modalities such as CT, MRI, ultrasound, and endoscopy, all of which are useful for localising lesions but are unable to distinguish between the tumour types (18). Endoscopic ultrasonography (EUS) can be used to identify morphological characteristics of tumours, such as the origin of tissue layer, the presence of a capsule, and if the mass contains cystic degeneration or internal haemorrhage. These can provide valuable diagnostic clues as to whether the mass is more likely to be a GIST or a desmoid tumour. However, there are no reliable sonographic features to differentiate GISTs from desmoid tumours and EUS is therefore also considered to be insufficient as a sole diagnostic modality (19, 20).

The key to diagnosis relies on pathology, including morphology, histology, and immunohistochemistry. Morphologically, GISTs are well-circumscribed tumours of varying size (0.5–8 cm on average but can be larger than 30 cm) and often accompanied by a pseudo-capsule containing hemorrhagic, necrotic, or cystic components (6, 13). Histologically, GISTs are commonly described as spindle cell tumours, although they can also be epithelioid, or more rarely, mixed spindle cell/epithelioid (1, 2, 13). GISTs are almost always CD117 positive on immunohistochemistry (95%), making this marker the gold standard for diagnosis. Diagnosis can be further confirmed by CD34 and DOG-1 positivity, as well as desmin and nuclear β-catenin negativity (3, 4, 7, 14, 21, 22). Desmoid tumours are quite different. Morphologically, these are described as firm, tan, and homogenous tumours without capsules and do not typically have necrotic or hemorrhagic features (13, 17, 23, 24). Histologically, desmoid tumours are described as spindle cell tumours with a denser, more collagenous stroma than seen in GISTs (11, 21). Majority of desmoid tumours have mutations in the APC/β-catenin pathway, resulting in nuclear β-catenin positivity on immunohistochemistry. They also tend to be desmin positive and CD117, CD34, and DOG-1 negative (12, 14, 21). In this case, although the patient’s morphological description on EUS that seemed to be in keeping with a GIST, histology demonstrated a low-grade spindle cell tumour and immunohistochemistry demonstrated CD117/CD34/DOG-1 negativity with patchy cytoplasmic and nuclear β-catenin staining, in keeping with desmoid fibromatosis.

The immunohistochemistry profile is of great importance when differentiating GISTs and desmoid tumours. However, it is important to consider that there may be overlap and mimicry between their immunohistochemistry profiles, specifically in cases where desmoid tumours are CD117 positive or GISTs are CD117 negative. It is also important to consider there are other non-epithelial gastrointestinal tumours that may share similarities to GISTs and desmoid tumours on immunohistochemistry profile, including leiomyomas, leiomyosarcomas, schwannomas, and inflammatory fibroid polyps (1, 25, 26). Therefore, thorough immunohistochemical staining to evaluate as many makers as possible is needed to ensure an appropriate diagnosis is being made. In addition to this, morphology and histology must be reviewed prior to confirming a diagnosis wherever possible (3, 13, 14).

The caveat of this case was that the initial biopsy sample could not differentiate a GIST from a desmoid tumour prior to exploratory laparotomy. As seen in this case, a high quality EUS-guided biopsy is not always feasible due to the location of the mass, and unsuccessful biopsy or partial sampling can lead to misdiagnosis. In many cases, the culprit mass must be surgically resected prior to undergoing more detailed pathology. Therefore, imaging modalities will be the diagnostic tool of choice prior to surgical intervention. The first line treatment in majority of cases is radical surgical excision (1, 3, 8, 11). As such, prior differentiation is not essential. However, the final diagnosis is important for prognostication and plan for ongoing medical management.

Differentiation of GISTs and desmoid tumours is key for proper prognostication. Desmoid tumours are considered benign and have a 5-year survival of 92%, despite being locally aggressive with a high risk of recurrence (11, 17, 27). GISTs, on the other hand, have “unknown malignant potential.” They have a relapse rate of 50% within 5 years and 10–25% of cases are metastatic (1, 2, 5). Differentiation between these tumours is also needed to determine the most appropriate second line therapy, should surgical resection fail or not be an option due to high risk of disfigurement or metastatic disease. For GISTs, the second line therapy of choice is CD117/KIT tyrosine kinase inhibitors, which have been shown to achieve stable disease or a partial response in up to 80% of patients (1–3). For desmoid tumours, second line therapy is often radiation, but can include other therapies such as chemotherapy and hormonal therapy, which have shown varying success (8, 12, 17, 24). Lastly, the distinction between tumours is paramount for timely investigation for associated diseases such as FAP in patients with desmoid tumours. Although desmoid tumours are rare in the general population, they are 850–1000 times more common in patients with FAP and may be found months to years preceding FAP diagnosis (8, 9, 16). Given a high morbidity and mortality rate secondary to colorectal cancer in FAP, all patients with desmoid tumours require further investigation with colonoscopy, as we did in our case (11, 15, 28).

## Conclusion

In summary, this case demonstrates that relying on imaging alone can result in misdiagnosis of a gastrointestinal tumour. Given the overlap and mimicry between gastrointestinal tumours, immunohistochemistry is needed to ensure an appropriate diagnosis is being made. Wherever possible, morphology and histology must also be reviewed to ensure correct diagnosis. Lastly, the distinction between non-epithelial gastrointestinal tumours is paramount for appropriate prognostication and management, including timely investigation for associated diseases such as FAP in patients with desmoid tumours.

## Data availability statement

The original contributions presented in the study are included in the article/supplementary material, further inquiries can be directed to the corresponding author.

## Ethics statement

Ethical review and approval was not required for the study on human participants in accordance with the local legislation and institutional requirements. The patients/participants provided their written informed consent to participate in this study. Written informed consent was obtained from the individual(s) for the publication of any potentially identifiable images or data included in this article.

## Author contributions

YG and LD identified the case as appropriate for write up and publication, and contributed to manuscript revision, read, and approved the submitted version. LA did the literature review and wrote all sections of the case report. JB and CM provided expertise on the pathology portion of the case report. J-EN provided expertise on the EUS portion of the case report. All authors were involved in the management of the patient in the case report.
